# Ruth Nussenzweig (1928–2018) Malaria Vaccine and Immunology Pioneer

**DOI:** 10.4269/ajtmh.18-1928

**Published:** 2018-07-02

**Authors:** Stephen L. Hoffman

**Affiliations:** Sanaria Inc., Rockville, Maryland

In 1967 Ruth Nussenzweig and colleagues at the New York University (NYU) School of Medicine reported in *Nature* that mice could be protected against rodent malaria by immunization with radiation attenuated sporozoites, the malaria parasite life cycle stage that is transmitted to humans by mosquitoes. Until publication of the 1967 paper there was little to no indication or hope that humans could be completely protected against malaria. Inspired by that 1967 report, by 1974 two groups working independently, one including NYU investigators, had shown that humans could be protected by exposure to mosquitoes carrying radiation attenuated *Plasmodium falciparum* (Pf) sporozoites.

In 1980 Dr. Nussenzweig, her husband Victor Nussenzweig, and colleagues, reported in *Science* and the *Journal of Experimental Medicine* that passive transfer of a monoclonal antibody to the major protein on the surface of sporozoites, the circumsporozoite protein (CSP), a protein they had discovered, protected mice against challenge with rodent malaria sporozoites. The 1980 papers laid the foundation for a scientific revolution in malaria molecular biology and vaccinology, which led 4 years later to independent publications by two groups, one from NYU, of the gene sequence of CSP, and 3 years after that to independent demonstrations by two groups, one from NYU, that immunization of humans with a recombinant protein or synthetic peptide vaccine, both entirely based on CSP, could protect against human malaria infection. Many years later, a recombinant protein vaccine based on CSP, called RTS,S, has completed Phase 3 clinical trials in Africa, and will soon be studied in pilot implementation trials in several hundred thousand infants in 3 countries in Africa. A vaccine based on radiation-attenuated Pf sporozoites, PfSPZ Vaccine, has shown significant protection in the U.S., Europe, and Africa, and will be assessed in Phase 3 clinical trials beginning in 2019. The vast majority of all humans ever protected against malaria by a vaccine have been protected with either PfCSP-based or Pf sporozoite-based vaccines, both developed through approaches that stem from the seminal Nussenzweig reports of 1967 and 1980.

Ruth Sonntag was born on June 20, 1928, in Vienna, Austria. Her parents, Barouch and Eugenia, were Jewish physicians. When Nazi Germany annexed Austria in 1938, her father was imprisoned. Shortly thereafter, while Ruth’s mother was waiting on line to visit her husband in prison, she was recognized by a prominent Austrian Nazi whom her father had helped previously. This man facilitated Barouch’s release from prison, and the family escaped on a harrowing train voyage to Belgium, where they hid until 1939, when they were able to go to Brazil. Ruth finished school in Brazil and studied medicine at the University of São Paulo (USP), where she met her classmate Victor Nussenzweig, who would become her husband, life companion, and scientific collaborator for more than 65 years; their marriage ceremony was in the Department of Parasitology library at the medical school in 1952. As medical students Ruth and Victor conducted research on *Trypansoma cruzi*, the cause of Chagas Disease. In a 1953 publication they reported that gentian violet destroyed *T. cruzi* in infected blood. Based on their work, gentian violet was adopted as preventative treatment of blood targeted for human transfusion in Brazil and other Latin American countries, an amazing contribution by medical students that foreshadowed what was to come.

In 1953 Dr. Nussenzweig finished medical school and became an Assistant Professor of Parasitology at USP. From 1958–1960 the Nussenzweigs were in Paris, where Ruth was a research fellow at the Laboratory of General Biochemistry, College de France. Another research fellowship in 1963 sent them to NYU. Intended to be a temporary relocation, it became permanent after a military coup in Brazil in 1964 made it impossible to continue to work at USP. In 1965 Dr. Nussenzweig became an Assistant Professor at NYU, and in 1972 she was promoted to Professor. In 1976 she became head of the Division of Parasitology in the Department of Microbiology, and in 1984 was appointed chair of the newly established Department of Medical and Molecular Parasitology at NYU, a position she held until 2002. Ruth Nussenzweig was the first woman ever to chair a department at the NYU School of Medicine.

While making the seminal findings described at the outset, Dr. Nussenzweig built and trained an incredible team of scientists at NYU, many of whom became internationally renowned leaders. Her NYU team systematically described the immunological mechanisms responsible for the protection induced by irradiated sporozoites, and created reagents used for studying the epidemiology of malaria in the field. Most of the early work focused on antibodies to sporozoites and specifically CSP. However, she and Victor subsequently led studies that also established roles for cellular immunity in mediation of protective immunity. She and her team trained scores of students and fellows from all over the world, many of whom became leaders in the field. This included many from Brazil, where Ruth, along with Victor, maintained scientific relations. Starting in 2012, she and Victor returned to do research in Brazil for some months each year, at the Federal University of São Paulo.

Ruth published her groundbreaking work in the most prestigious scientific journals. For example, in 1967, 1968, 1969, and 1970 she published scientific articles in *Nature*, and in total she published more than 230 scientific papers. She published 22 articles in the *American Journal of Tropical Medicine and Hygiene*. She received numerous awards in the U.S., Europe, and Brazil, including the Paul-Ehrlich and Ludwig Darmstaedter Prize (Germany); the Joseph Augustin LePrince Medal, in recognition of outstanding work in the field of malariology from the American Society of Tropical Medicine and Hygiene (ASTMH) in 1997; the Denis Thienpont Prize from the Royal Academy of Medicine of Belgium; the Gold Medal from the Sabin Vaccine Institute; the Warren Alpert Foundation Award from Harvard; and most recently in 2017, the inaugural Clara Southmayd Ludlow Medal of the ASTMH, named for a woman leader in the field of tropical medicine. Ruth was an active member of the ASTMH, which she joined in 1965. She was selected as a member/fellow of numerous honorific societies including the Royal Academy of Medicine of Belgium, the Brazilian Academy of Sciences, the American Academy of Arts and Sciences, the National Academy of Medicine (USA), and National Academy of Science (USA).

Ruth Sonntag Nussenzweig was a unique force, a visionary, a leader, and a brilliant scientist, who made her mark despite having to move at critical times in her formative years from Nazi-ruled Austria and then from military-dominated Brazil to the United States. Ruth Nussenzweig was a pioneer, whose efforts provided the foundation for the development of malaria vaccines now on the horizon, to help rid the world of a disease that still causes illness in over 200 million and death in a half million people each year. She is survived by her husband, Victor, her three children, Michel, Sonia, and Andre, all highly successful and renowned academics undoubtedly inspired by their mother (and father), and six grandchildren. Dr. Nussenzweig will be greatly missed, but she lives on through her prodigious scientific contributions and her many students, trainees, and colleagues who continue to contribute valuable tropical medicine research.

**Figure:**
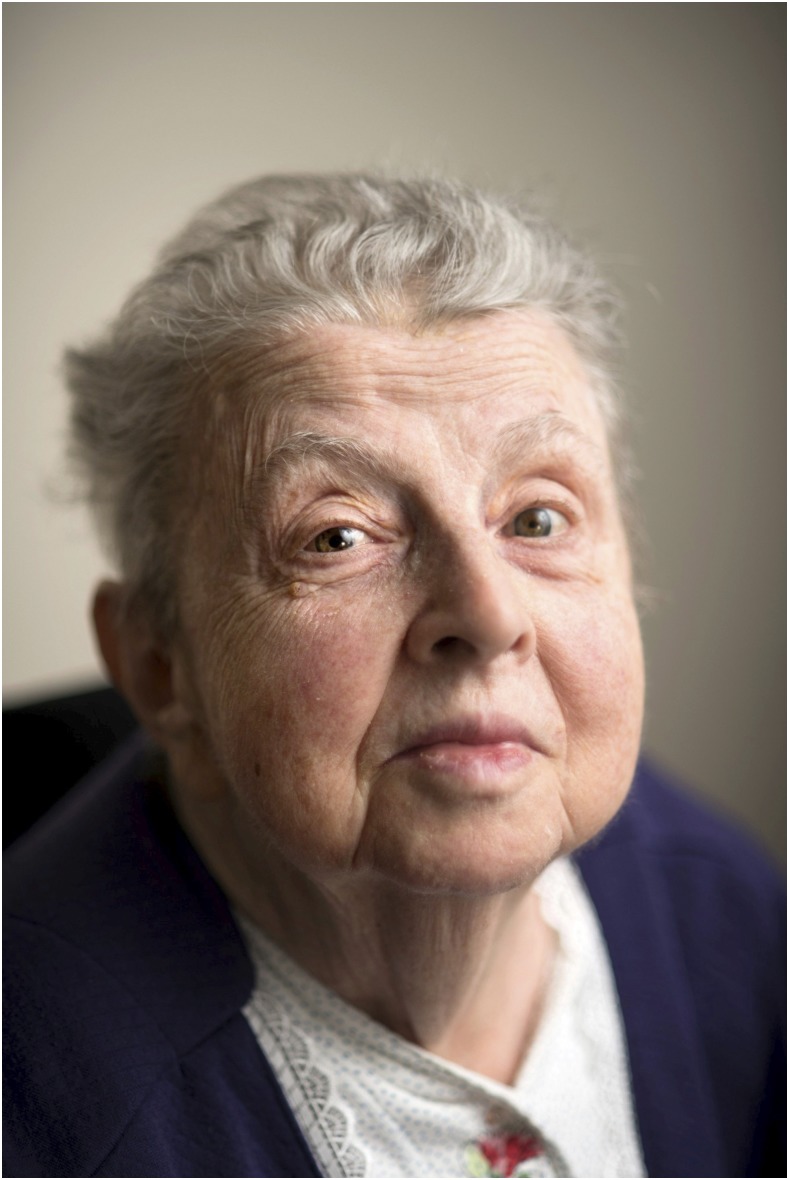
Ruth S. Nussenzweig, MD, PhD

**Figure:**
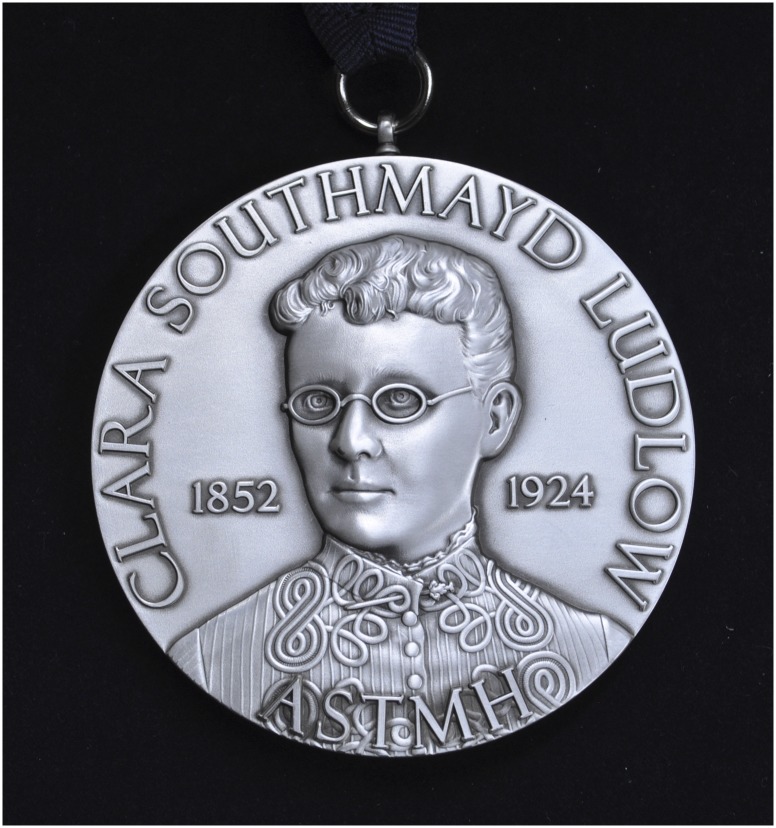


**Figure:**
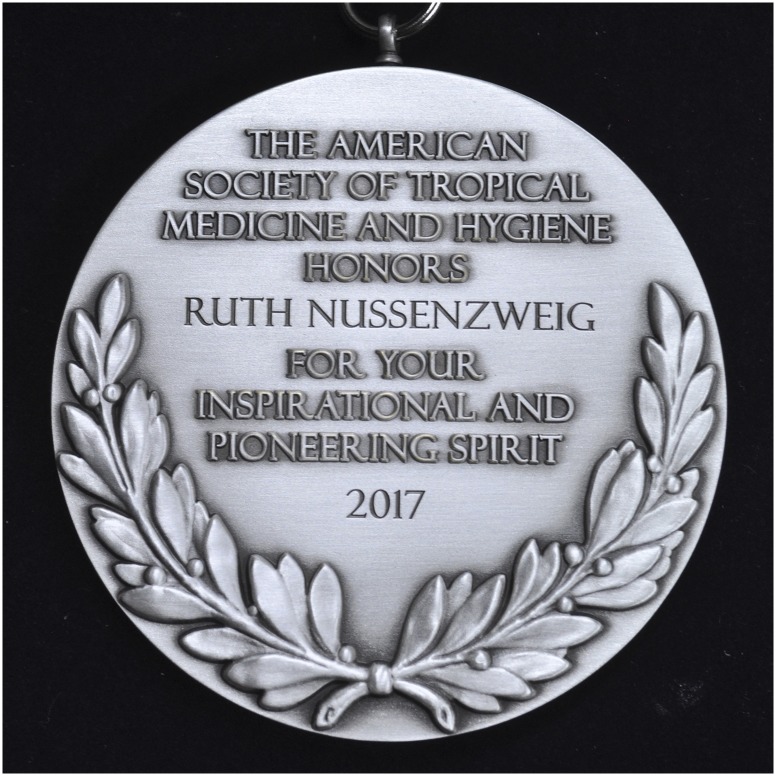
Dr. Nussenzweig was the first recipient of ASTMH’s Clara Southmayd Ludlow Medal.

